# Epigenetic and metabolic reprogramming via nanotechnology: a synergistic approach to cancer vaccination in lung tumors

**DOI:** 10.3389/fgene.2025.1666561

**Published:** 2025-10-08

**Authors:** Min Xu, Xialin Zhang, Xinlin Yu, Cheng Ma, Xinwei Li, Gang Chen, Gang Yuan, Sheng Lin, Ran Cui

**Affiliations:** ^1^ Department of Respiratory and Critical Care Medicine, Affiliated Hospital of Chengdu University, Chengdu, Sichuan, China; ^2^ Department of Oncology, Affiliated Hospital of Southwest Medical University, Luzhou, Sichuan, China; ^3^ Department of Oncology, Affiliated Hospital of Chengdu University, Chengdu, Sichuan, China; ^4^ Department of Respiratory and Critical Care Medicine, First People’s Hospital of Neijiang, Neijiang, Sichuan, China; ^5^ Department of General Surgery (Hepatopancreatobiliary Surgery), Affiliated Hospital of Southwest Medical University, Luzhou, Sichuan, China; ^6^ Department of Interventional & Vascular, Affiliated Traditional Chinese Medicine Hospital of Southwest Medical University, Luzhou, Sichuan, China

**Keywords:** metabolic reprogramming, epigenetic regulation, nanoplatforms, cancer vaccines, immunosuppression, lung tumors

## Abstract

Cancer vaccines represent a promising therapeutic modality in immuno-oncology, yet their efficacy is severely constrained within the immunosuppressive microenvironment of lung tumors. Metabolic reprogramming and epigenetic dysregulation are now understood as critical, interconnected determinants that orchestrate tumor microenvironment (TME) immunosuppression and fundamentally shape anti-tumor immune responses. This review comprehensively examines the mechanistic interplay between metabolic reprogramming and epigenetic regulation, and how nanoplatform technologies can be engineered to modulate these axes to augment cancer vaccine efficacy. We analyze advanced nano-delivery system design strategies, the synergistic effects of combining metabolic intervention with epigenetic modification, and their application in overcoming the formidable barriers of the lung TME. By integrating recent advances in nanotechnology, epigenetics, and tumor immunometabolism, we provide critical insights into the development of next-generation cancer vaccines. Furthermore, we propose a novel conceptual framework—The Epi-Met-Immune Synergistic Network—to dissect these interactions and identify key nodes for rational therapeutic intervention, aiming to enhance and sustain durable anti-tumor immunity.

## Introduction

The landscape of cancer immunotherapy has been revolutionized by the advent of cancer vaccines, which harness the host immune system’s capacity to mount specific anti-tumor responses (Sharma et al., 2017). Despite remarkable clinical successes in certain malignancies, lung tumors present formidable challenges due to their profoundly immunosuppressive microenvironment (Looi et al., 2019). The intricate interplay between metabolic dysregulation and epigenetic alterations within the tumor microenvironment (TME) orchestrates a complex network of immunosuppressive mechanisms that severely compromise vaccine efficacy (Bader et al., 2020).

At its core, cancer is a disease of dysregulated gene expression, driven not only by genetic mutations but also by profound epigenetic alterations. Epigenetic reprogramming—encompassing DNA methylation, histone modifications, and chromatin remodeling—serves as a fundamental mechanism by which tumor cells evade immune surveillance and sustain proliferation across a wide spectrum of malignancies. During the escape phase of cancer immunoediting, genetic and epigenetic alterations in tumor cells—reversible through nanomaterial interventions—result in tumor antigen deficiency and impaired antigen-presenting machinery. These changes also foster the development of an immunosuppressive tumor microenvironment, characterized by expanded populations of immunosuppressive cells and accumulated immunosuppressive molecules, which collectively inactivate cytotoxic immune cells such as cytotoxic T lymphocytes (CTLs) (Liu et al., 2024). The strategic reversal of these epigenetic aberrations has thus emerged as a promising therapeutic avenue in oncology, aiming to restore antitumor immunity and sensitize tumors to various immunotherapies.

This promise, however, remains largely unrealized specifically in lung cancer, where the therapeutic vaccines have consistently faltered against a profoundly immunosuppressive microenvironment (Lahiri et al., 2023). Here, the intricate interplay between metabolic dysregulation and the aforementioned epigenetic alterations within the TME orchestrates a complex network of immunosuppressive mechanisms that severely compromise vaccine efficacy (Giaccone et al., 2015). Landmark trials underscore this difficulty: the MAGE-A3 protein vaccine, despite successfully inducing antigen-specific T-cell responses, failed to improve overall or disease-free survival and the L-BLP25 vaccine similarly did not show significant difference in overall survival in Phase III studies (Batchu et al., 2014; Butts et al., 2014). These data highlight a critical challenge: the TME orchestrates a complex network of immunosuppressive mechanisms, driven by factors such as the accumulation of regulatory T cells, that actively subverts vaccine-induced immunity and severely compromises therapeutic efficacy.

Metabolic reprogramming in lung tumors encompasses a spectrum of alterations, including enhanced glycolysis, aberrant amino acid metabolism, and lipid metabolic rewiring, collectively creating a hostile metabolic milieu that impairs immune cell function (Arner and Rathmell, 2023). Concurrently, epigenetic modifications—including DNA methylation patterns, histone post-translational modifications, and chromatin remodeling—fundamentally alter gene expression programs that govern immune recognition and response (Dai et al., 2021). These epigenetic changes not only affect tumor cells but also reprogram infiltrating immune cells, establishing durable immunosuppressive phenotypes (Cao and Yan, 2020).

The emergence of nanotechnology has opened unprecedented opportunities for precision medicine approaches that simultaneously target metabolic and epigenetic abnormalities (Zhang et al., 2023). Nanoplatforms offer unique advantages including enhanced drug stability, targeted delivery, controlled release kinetics, and the capacity for multi-drug co-delivery (Zhang et al., 2021). By integrating metabolic modulators with epigenetic therapeutics within sophisticated nanocarriers, it becomes possible to synergistically reprogram the TME and potentiate cancer vaccine responses (Ren et al., 2023).

This review provides a comprehensive analysis of how epigenetic-regulated nanoplatforms influence metabolic reprogramming to enhance cancer vaccine efficacy. We examine the molecular mechanisms underlying TME immunosuppression, evaluate current nanoplatform design strategies, and discuss emerging therapeutic approaches. Critically, we introduce the Epi-Met-Immune Synergistic Network as a conceptual model to deconstruct the complex feedback loops that drive therapeutic resistance, thereby providing a rational basis for designing multi-targeted nanoplatforms to overcome these barriers.

## Characteristics of the immunosuppressive lung TME and challenges for cancer vaccines

### Immunosuppressive mechanisms in the lung tumor microenvironment

The lung tumor microenvironment represents a paradigm of immune dysfunction, characterized by multiple interconnected immunosuppressive mechanisms (Altorki et al., 2019). Regulatory T cells (Tregs) accumulate within lung tumors through chemokine-mediated recruitment and local expansion, establishing dominant immunosuppressive networks (Togashi et al., 2019). These Tregs express high levels of checkpoint molecules including CTLA-4 and PD-1, while secreting immunosuppressive cytokines such as IL-10 and TGF-β (Zhang et al., 2024). The functional stability of intratumoral Tregs is maintained through specific metabolic adaptations, including enhanced fatty acid oxidation and resistance to lactate-induced suppression (Zhou L. et al., 2024).

Myeloid-derived suppressor cells (MDSCs) represent another critical component of lung tumor immunosuppression (He et al., 2025). These heterogeneous populations of immature myeloid cells accumulate in response to tumor-derived factors including GM-CSF, VEGF, and prostaglandins (Li K. et al., 2021). MDSCs employ multiple mechanisms to suppress anti-tumor immunity, including arginine depletion through arginase-1 expression (Bronte et al., 2003), production of reactive oxygen species, and induction of Treg differentiation (Groth et al., 2019; Serafini et al., 2006). The metabolic profile of MDSCs is characterized by enhanced glycolysis and altered lipid metabolism, which not only supports their immunosuppressive functions but also renders them resistant to metabolic stress within the TME (Jin et al., 2023; Yan et al., 2019).

**FIGURE 1:**
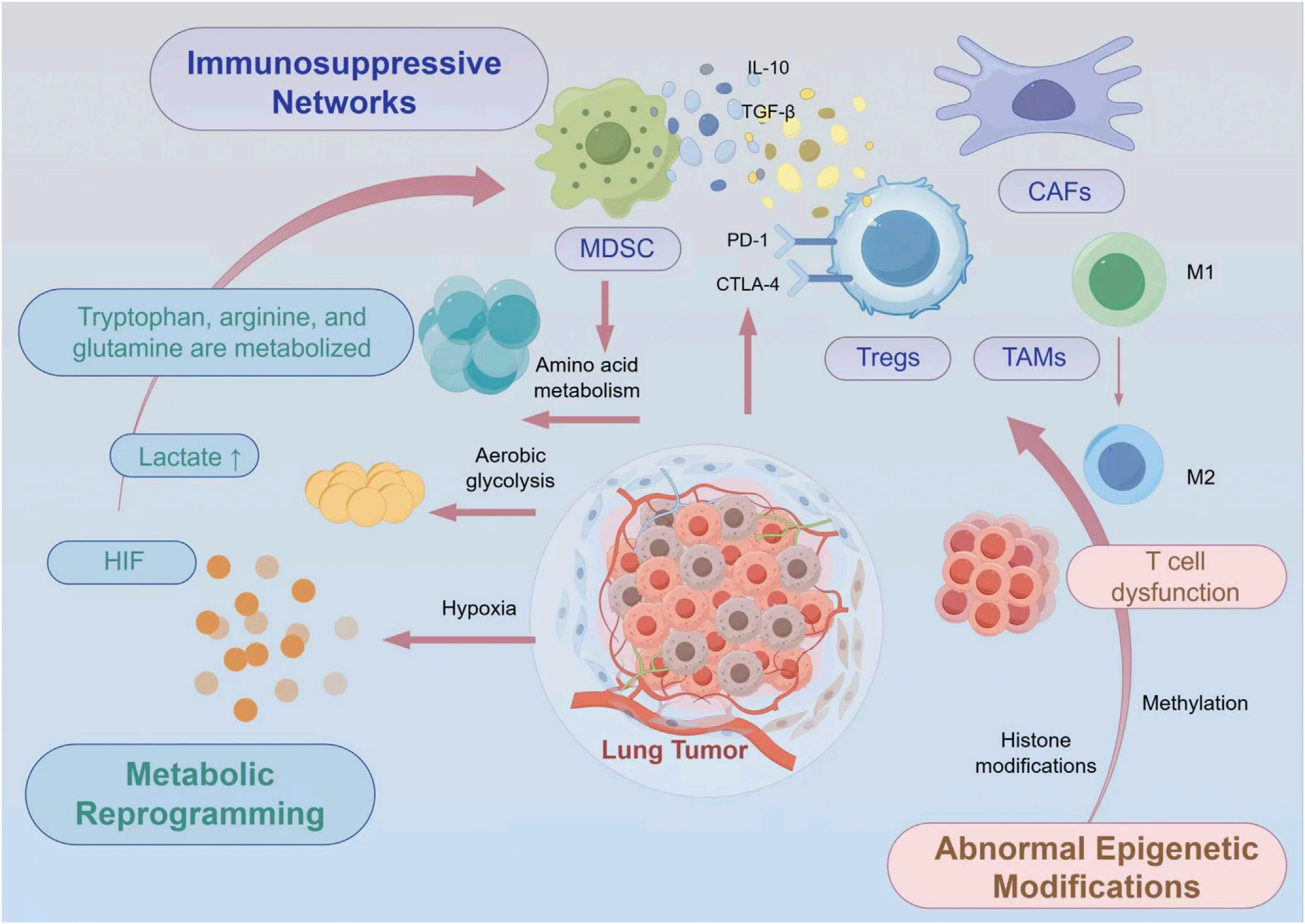
Comprehensive Schematic Illustration of the Immunosuppressive Lung Tumor Microenvironment. The lung tumor microenvironment exhibits pronounced immunosuppressive characteristics, including the accumulation and functional activation of immunosuppressive cells such as Tregs and MDSCs, alongside aberrant expression of immune checkpoint molecules including PD-L1. Concurrently, metabolic abnormalities (lactate accumulation, hypoxia, amino acid depletion) and epigenetic dysregulation (T cell exhaustion, immune cell epigenetic reprogramming) further reinforce these immunosuppressive networks. These multifaceted mechanisms collectively present significant challenges for the application of cancer vaccines, necessitating integrated therapeutic approaches that simultaneously address these interconnected immunosuppressive pathways.

The expression of immune checkpoint molecules extends beyond infiltrating immune cells to encompass tumor cells and stromal components. Lung tumors frequently upregulate PD-L1 expression through various mechanisms including oncogenic signaling, inflammatory cytokines, and hypoxia-inducible factors (Yamaguchi et al., 2022). Additionally, alternative checkpoint pathways such as TIM-3, LAG-3, and TIGIT create redundant immunosuppressive networks that limit vaccine-induced immune responses (Cai et al., 2023) (Figure 1).

### Metabolic abnormalities and their impact on immune cell function

The metabolic landscape of lung tumors profoundly shapes immune cell function and fate. Aerobic glycolysis in tumor cells leads to excessive lactate production, creating an acidic microenvironment that impairs T cell proliferation and cytotoxic function (Wu et al., 2023). Lactate acts as both a metabolic substrate and signaling molecule, promoting regulatory T cell differentiation while suppressing effector T cell responses (Llibre et al., 2025). The acidic pH also interferes with antibody-dependent cellular cytotoxicity and reduces the efficacy of therapeutic antibodies (Liu Y. et al., 2022).

Hypoxia represents another hallmark of the lung tumor metabolic environment. Regions of severe hypoxia stabilize hypoxia-inducible factors (HIFs), which orchestrate transcriptional programs that promote immunosuppression (Lee et al., 2020). HIF-1α drives the expression of checkpoint ligands, enhances MDSC recruitment, and promotes the differentiation of tumor-associated macrophages toward immunosuppressive phenotypes (Luo et al., 2022). Moreover, hypoxia impairs dendritic cell maturation and antigen presentation, critical processes for effective cancer vaccine responses (Kheshtchin et al., 2016).

Amino acid metabolism within lung tumors creates additional immunosuppressive barriers (Chen et al., 2024). Tumor cells and immunosuppressive myeloid cells deplete essential amino acids including tryptophan, arginine, and glutamine from the microenvironment (Kheshtchin et al., 2016). Tryptophan catabolism through indoleamine 2,3-dioxygenase (IDO) produces kynurenine metabolites that directly suppress T cell proliferation and promote Treg differentiation (Munn et al., 1998; Fallarino et al., 2006). Arginine depletion by arginase-expressing MDSCs impairs T cell receptor signaling and memory formation, while glutamine restriction compromises T cell activation and effector function (Crump et al., 2021).

### Epigenetic determinants of T cell exhaustion in the lung tumor microenvironment

T cell exhaustion in lung tumors represents a paradigmatic example of epigenetically-encoded immune dysfunction that profoundly limits cancer vaccine efficacy (Pan and Zheng, 2020). This functionally impaired state is not merely transient but is stabilized through comprehensive epigenetic reprogramming, establishing a self-reinforcing gene expression program resistant to conventional immunotherapeutic interventions (Belk et al., 2022a). Genome-wide epigenetic profiling has revealed distinctive chromatin accessibility landscapes in exhausted tumor-infiltrating T cells, characterized by inaccessible chromatin at effector gene loci juxtaposed with enhanced accessibility at inhibitory receptor genes (Belk et al., 2022b). These alterations are accompanied by region-specific DNA methylation patterns, including hypermethylation at cytokine promoters (IFN-γ, TNF-α) and hypomethylation at immune checkpoint loci (PD-1, CTLA-4), collectively restricting T cell functional plasticity (Smith et al., 2020).

The epigenetic architecture of exhausted T cells is further sculpted by a characteristic histone modification signature. Enhancer regions of effector genes display reduced H3K27ac and H3K4me1, while inhibitory receptor loci exhibit enrichment of these activation-associated marks. Concurrently, the repressive H3K27me3 mark, catalyzed by the PRC2 complex via EZH2, accumulates at critical effector gene promoters, silencing cytotoxic programs while sparing checkpoint receptor expression. This role of EZH2 as a key enforcer of immunosuppression is not limited to exhausted T cells; forryptophan catabolism through indoleamine 2,3-dioxygenase (IDO) produces kynurenine metabolites that directly suppress T cell proliferation and promote Treg differentiation instance, recent work demonstrates that its hyperactivation in regulatory T cells also enhances their suppressive capacity and stability (Peeters et al., 2024). This epigenetic imbalance is perpetuated by increased HDAC activity, which depletes activating acetylation marks at key effector loci, diminishing both their transcriptional potential and functional capacity (Ibrahim et al., 2024). Recent analyses have further revealed that these modifications are progressively established during tumor progression, creating increasingly fixed states of dysfunction that correspond with resistance to checkpoint blockade therapy (Perrier et al., 2020).

The architecture of immunosuppression in the lung TME is fortified by the epigenetic reprogramming of multiple immune lineages, creating formidable barriers to cancer vaccine efficacy. Beyond the tumor cells themselves, key innate immune cells are functionally subverted; TAMs are skewed towards an M2-like phenotype through alterations in their enhancer landscapes, while MDSCs are locked into an immature, suppressive state by stable epigenetic modifications (Niu et al., 2022). This intricate cellular network, however, exposes critical gaps in our fundamental understanding. For instance, the precise molecular pathways through which tumor-derived metabolites like adenosine and reactive oxygen species (ROS) drive epigenetic silencing in effector CD8^+^ T cells remain to be fully elucidated (Yerinde et al., 2019). Furthermore, it is unclear how distinct inhibitory axes—such as immune checkpoint over-activation, HLA-I downregulation, and metabolic hostility—synergize to create a composite barrier that is impenetrable to single-antigen vaccines (Yi et al., 2019). Finally, the dynamic interplay between evolving tumor antigen heterogeneity and the progressive exhaustion of immune cells represents a core clinical bottleneck, and whether personalized antigen design can reverse this tolerance to establish long-term memory remains a pivotal unanswered question (Jia et al., 2022). Herein lies the central challenge and opportunity: because these immunosuppressive states are epigenetically encoded, targeting the epigenetic machinery itself offers a foundational strategy to dismantle the entire network and overcome the core barriers limiting cancer vaccine efficacy.

## The role of epigenetic regulation in reversing lung cancer immunosuppression

### DNA methyltransferase inhibitors promoting lung cancer antigen expression

DNA methyltransferase inhibitors (DNMTi) have emerged as powerful tools for reversing epigenetic silencing in lung tumors (Goyal et al., 2023; Vendetti and Rudin, 2013). Aberrant DNA hypermethylation silences numerous tumor antigens, including cancer-testis antigens (CTAs), neoantigens, and MHC class I molecules, thereby limiting tumor immunogenicity (Geissler et al., 2024). DNMTi treatment induces global DNA hypomethylation, reactivating silenced tumor antigens and enhancing immune recognition.

The mechanisms of DNMTi-mediated immune activation extend beyond simple antigen re-expression (Huang et al., 2021). DNMTi treatment activates endogenous retroviral elements and repetitive sequences, triggering viral mimicry responses. This phenomenon induces type I interferon signaling through activation of cytosolic nucleic acid sensors, creating an inflammatory milieu that enhances dendritic cell activation and T cell priming (Chiappinelli et al., 2015). Furthermore, DNMTi treatment upregulates antigen processing and presentation machinery, including TAP transporters, immunoproteasome subunits, and MHC molecules (Ignatz-Hoover et al., 2022).

Recent studies have demonstrated that DNMTi can reprogram the metabolic landscape of lung tumors. By altering the methylation status of metabolic gene promoters, DNMTi treatment reduces glycolytic flux and lactate production, partially alleviating metabolic immunosuppression (Xu et al., 2023). Additionally, DNMTi-induced changes in tumor cell metabolism can enhance their susceptibility to immune-mediated killing through metabolic checkpoint mechanisms (Wang et al., 2025).

### Histone deacetylase inhibitors enhancing T cell memory responses

HDAC inhibitors represent another class of epigenetic modulators with significant immunomodulatory potential (Hicks et al., 2020). In the context of lung cancer immunotherapy, HDAC inhibitors exert pleiotropic effects that enhance vaccine-induced immune responses (Li X. et al., 2021). By increasing histone acetylation at memory-associated gene loci, HDAC inhibitors promote the differentiation and maintenance of memory T cells, crucial for durable anti-tumor immunity (Ellmeier and Seiser, 2018).

HDAC inhibition in T cells enhances the expression of transcription factors associated with memory formation (Ibrahim et al., 2024; Montacchiesi and Pace, 2022). These transcriptional changes are accompanied by metabolic reprogramming toward oxidative phosphorylation, a metabolic profile that supports memory T cell survival and function. Moreover, HDAC inhibitors reduce the expression of inhibitory receptors on T cells, potentially reversing exhaustion phenotypes and restoring effector function.

The effects of HDAC inhibitors extend to antigen-presenting cells, where they enhance costimulatory molecule expression and cytokine production. Dendritic cells treated with HDAC inhibitors show improved antigen presentation capacity and increased production of T cell-polarizing cytokines (De Sa Fernandes et al., 2024). In tumor-associated macrophages, HDAC inhibition can shift polarization away from immunosuppressive M2-like phenotypes toward inflammatory M1-like states (Yang et al., 2025).

### Non-coding RNAs in immune regulation

The landscape of non-coding RNAs, including microRNAs (miRNAs) and long non-coding RNAs (lncRNAs), represents an emerging frontier in epigenetic immunomodulation. In lung tumors, specific miRNA signatures regulate key aspects of immune function (Zhu et al., 2021; Iqbal et al., 2019). Tumor-derived exosomes carrying immunosuppressive miRNAs, such as miR-21 and miR-155, can reprogram immune cells toward tolerogenic phenotypes (Shokati and Safari, 2023; Yang et al., 2013). Conversely, certain miRNAs function as tumor suppressors and immune activators, suggesting therapeutic potential for miRNA-based interventions (Kim and Croce, 2023).

LncRNAs orchestrate complex regulatory networks that influence immune responses at multiple levels. The lncRNA NEAT1 promotes MDSC expansion and function, while MALAT1 regulates dendritic cell differentiation and antigen presentation (Wu et al., 2018). Targeting these lncRNAs through antisense oligonucleotides or CRISPR-based approaches offers novel strategies for modulating tumor immunity (Arun et al., 2018).

Recent advances in understanding circular RNAs (circRNAs) have revealed their roles in immune regulation. CircRNAs can function as miRNA sponges, modulating the availability of miRNAs that regulate immune checkpoints and inflammatory responses (Meng et al., 2024). The stability and tissue-specific expression of circRNAs make them attractive targets for immunomodulatory interventions (Misir et al., 2022).

**FIGURE 2:**
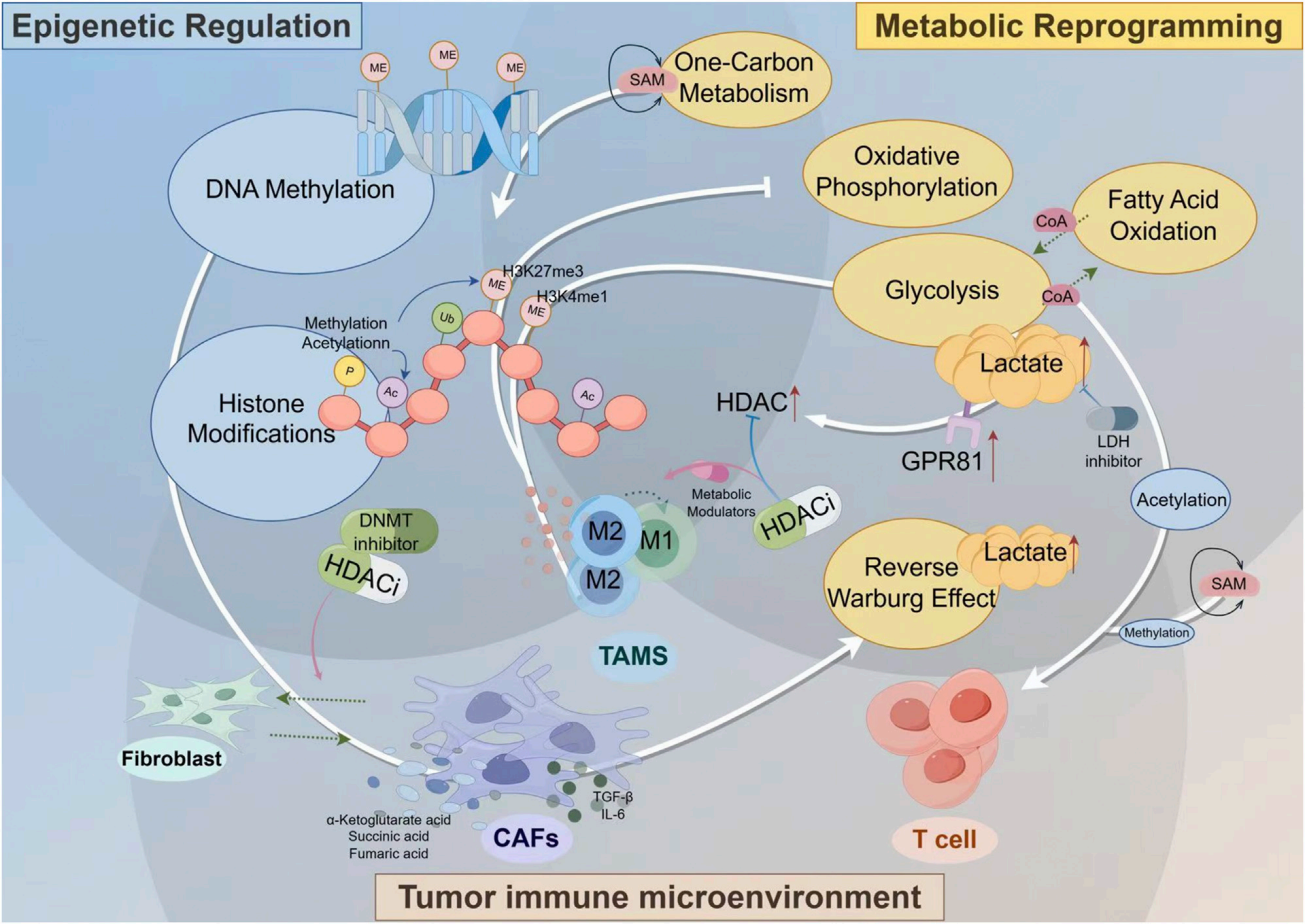
The Interplay of Metabolic Reprogramming and Epigenetic Regulation in the Tumor Immune Microenvironment. Key stromal cells, such as CAFs and TAMs, adopt altered metabolic programs like the Reverse Warburg Effect, producing an abundance of metabolites including lactate, SAM, and Acetyl-CoA. These molecules are not merely metabolic byproducts but act as critical signaling molecules (for example lactate via its GPR81 receptor to upregulate HDAC) and essential substrates for epigenetic enzymes that control DNA methylation and histone modifications. This direct metabolic-epigenetic link ultimately dictates the functional phenotype of immune cells, crucially promoting the polarization of TAMs towards an immunosuppressive M2 state. The diagram highlights how this self-reinforcing cycle can be therapeutically targeted with inhibitors for key nodes like HDAC, DNMT, and LDH, offering a strategy to break the cycle and reprogram the TME towards an anti-tumor state.

While these epigenetic modulators—DNMT inhibitors, HDAC inhibitors, and non-coding RNA-based therapeutics—offer powerful, mechanistically distinct avenues to reverse immunosuppression, their translation into effective clinical strategies for lung cancer is hampered by a series of profound and interconnected challenges. The suppressive TME, densely populated by Tregs and MDSCs, remains a formidable barrier that can neutralize the benefits of vaccine-induced T cells (Munn and Bronte, 2016). Furthermore, the technical hurdles in developing truly personalized neoantigen vaccines, from prediction accuracy to overcoming tumor heterogeneity, limit broad applicability (Wu et al., 2024). Finally, the systemic delivery of these potent agents raises significant concerns regarding off-target effects and toxicity, highlighting a critical need for delivery systems that can precisely target tumor tissue while protecting the payload (Manzari et al., 2021). Therefore, simply possessing these epigenetic tools is not enough; the central challenge lies in deploying them effectively within the complex biological landscape of the tumor. This necessitates the development of advanced delivery platforms capable of integrating multiple therapeutic strategies to dismantle the immunosuppressive network at its core (Figure 2).

## The Epi-Met-Immune synergistic network: a framework for therapeutic design

### Beyond linear pathways: the need for an integrated network model

Designing therapies for the lung TME demands a departure from linear thinking. The intertwined challenges of epigenetic silencing and metabolic hostility are not independent pathways but are locked in a profound bidirectional interplay, creating pathological feedback loops that establish and maintain a remarkably stable immunosuppressive state. A metabolic alteration, for example, can drive an epigenetic change that, in turn, transcriptionally reinforces the aberrant metabolic phenotype and its downstream immunological consequences (Lu and Thompson, 2012). To deconstruct this complexity and move beyond empirical drug combinations, we propose the Epi-Met-Immune Synergistic Network, a multi-layered framework designed to map the key molecular and cellular players, their dynamic interactions, and the critical nodes for rational therapeutic intervention.

### Layers and nodes of the network

The network is conceptualized as three interconnected, interdependent layers. The foundational Epigenetic Layer comprises the architects of the chromatin landscape—the enzymatic machinery that writes, erases, and reads epigenetic marks. Key nodes here include DNMTs, HDACs, and histone methyltransferases like EZH2. This epigenetic control directly governs the Metabolic Layer, which comprises core metabolic pathways whose effector metabolites function dually as cellular fuel and potent signaling molecules (Keating and El-Osta, 2015). Critical nodes include glycolysis and oxidative phosphorylation (OXPHOS), while metabolites such as lactate, succinate, and α-ketoglutarate directly influence epigenetic enzyme activity, and the availability of universal donors like S-adenosylmethionine (SAM) and Acetyl-CoA links metabolic status directly back to epigenetic potential (Yu and Li, 2024; Yellen, 2018). Ultimately, the functional output of this intricate epi-metabolic crosstalk manifests in the Immune Layer, which encompasses the primary cellular actors of the anti-tumor response, including cytotoxic effector cells (CD8^+^ T cells, NK cells), immunosuppressive populations (Tregs, MDSCs, M2-polarized TAMs), and professional antigen-presenting cells (DCs).

### Dynamic interactions and paradigmatic feedback loops

**FIGURE 3:**
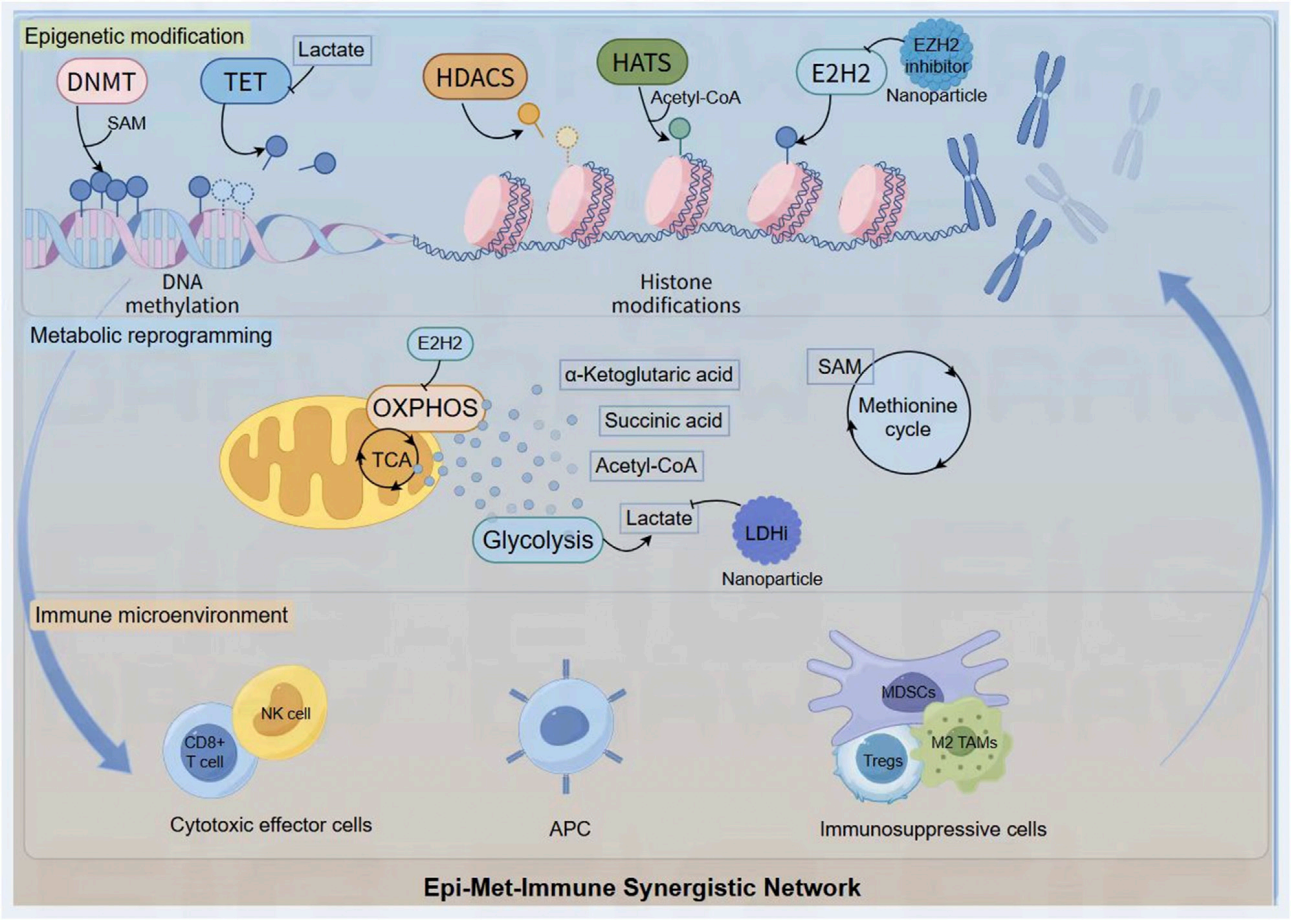
The Epi-Met-Immune Synergistic Network. Key metabolites generated from pathways like glycolysis and the methionine cycle (for example lactate, Acetyl-CoA, SAM) act as critical cofactors or inhibitors for epigenetic enzymes (DNMT, TET, HATs), directly linking the cell’s metabolic state to the regulation of DNA and histone modifications. This interplay is bidirectional, as epigenetic regulators like EZH2 can in turn control metabolic programs such as OXPHOS. This self-reinforcing feedback loop ultimately shapes the immune landscape, promoting a shift from cytotoxic effector cells to a dominant population of immunosuppressive cells (Tregs, MDSCs, M2 TAMs). The network serves as a rational blueprint for therapeutic intervention, where nanoplatforms are designed to deliver inhibitors against key nodes (for example EZH2i, LDHi) to simultaneously disrupt these pathological circuits and dismantle the foundations of tumor immunosuppression.

The true power of this framework lies in mapping the self-perpetuating circuits that drive therapeutic resistance. A paradigmatic example is the Warburg effect, which results in a lactate-rich TME (Lane et al., 2020). Lactate, now understood to be a potent oncometabolite, acts as a competitive inhibitor of α-ketoglutarate-dependent dioxygenases, including TET enzymes and certain histone demethylases (Faubert et al., 2017). This epigenetic reprogramming cripples the expression of key effector cytokines like IFN-γ and granzyme B in infiltrating CD8^+^ T cells, thus directly linking a metabolic byproduct to profound immune dysfunction via an epigenetic mechanism (Zebley et al., 2020). This crosstalk is profoundly bidirectional. Conversely, epigenetic programs can dictate metabolic fate, as seen in a T cell destined for exhaustion. Here, key epigenetic writers like EZH2 actively enforce a repressive transcriptional program, silencing entire gene networks required for T-cell proliferation, survival, and metabolic fitness (Zhao et al., 2016). This entire pathological loop is stabilized by the hypoxic, nutrient-poor conditions of the TME, creating a state of immune paralysis that is remarkably resistant to reversal (Figure 3).

### Network plasticity and therapeutic sequencing

Crucially, the Epi-Met-Immune Network is not a static entity but a dynamic system that evolves under selective pressure. In nascent tumors, the network’s connections may be highly plastic and malleable, representing a state of reversible immunosuppression. However, under the relentless pressure of tumor progression and therapy, these connections can become progressively hardwired or canalized. This is exemplified by T-cell exhaustion, where initial, reversible dysfunction (plasticity) transitions into a deeply entrenched epigenetic state (fixation) that is profoundly resistant to reversal by conventional immunotherapies (Blank et al., 2019).

This temporal evolution is not merely a challenge; it presents a critical, yet largely unexplored, therapeutic opportunity: the strategic sequencing of interventions. The concept of a fixed immunosuppressive state raises pivotal questions for clinical trial design. Can epigenetic modulators be deployed as priming agents to reverse epigenetic fixation and reopen a window of vulnerability to subsequent cancer vaccines or ICIs? What is the optimal duration and timing of this window before the network re-establishes its resilient, immunosuppressive state? Therefore, understanding the network’s temporal dynamics is paramount for designing therapies that are not only potent but also precisely timed to exploit moments of maximum vulnerability, a concept we term chronotherapy in this context.

### A rational map for nanotherapeutic intervention

Crucially, the Epi-Met-Immune Network serves as a rational map for a paradigm shift in therapeutic design—from single-agent targeting to multi-pronged, systems-level disruption. By visualizing the interconnected nodes, we can identify strategic points for intervention. A nanoplatform delivering an EZH2 inhibitor targets a key node in the Epigenetic Layer, while another carrying a lactate dehydrogenase inhibitor (LDHi) severs a critical link in the Metabolic Layer.

The ultimate goal, uniquely enabled by advanced nanoplatforms, is to co-deliver multiple agents that simultaneously attack different pathological connections within this network. This represents a move towards the controlled demolition of the entire immunosuppressive architecture. Nanocarriers are the essential enabling technology for this strategy, as they can ensure that distinct therapeutic agents arrive at the same tumor site at the same time, a prerequisite for disrupting a tightly regulated biological network. This systems-level approach offers a far more robust strategy to dismantle the foundations of immunosuppression and unlock the full potential of cancer vaccines.

### Epigenetic influence on metabolic reprogramming in enhancing lung cancer vaccine response

The functional manifestation of the Epi-Met-Immune Network is profoundly governed by its spatial organization within the tumor architecture. The TME is not a homogenous mixture of cells and metabolites; rather, it is a structured landscape with distinct ecological niches that dictate the network’s local topology. In the tumor’s hypoxic core, for instance, the network is dominated by metabolic suppression, where HIF-1α activation drives intense glycolysis, lactate accumulation, and subsequent epigenetic silencing of T-cell effector programs. In stark contrast, at the invasive front or proliferative margin, where immune infiltration is more active, the network may be rewired to favor adaptive resistance mechanisms, such as IFN-γ-driven PD-L1 expression and T-cell exhaustion mediated by direct cell-cell contact.

Unveiling this spatial heterogeneity is no longer a theoretical exercise but a tangible goal, enabled by the advent of Spatial Omics. Technologies like spatial transcriptomics and metabolomics are beginning to provide high-resolution maps of the network’s activity, moving our understanding from bulk analysis to a spatially resolved atlas. This granular view is critical, as it provides the ultimate rationale for developing “smart” nanoplatforms capable of navigating to and responding within specific microenvironmental niches (for example hypoxia, acidity). Therefore, the following analysis of individual cell populations must be interpreted through this spatial lens, recognizing that their epigenetic and metabolic states are fundamentally shaped by their precise location within the tumor ecosystem.

### Epigenetic regulation of lung cancer-associated macrophages

TAMs in lung cancer undergo extensive epigenetic reprogramming that shapes their metabolic and functional phenotypes (Morrissey et al., 2021). The transcription factor landscape of TAMs is fundamentally altered through changes in enhancer accessibility and promoter methylation (Larionova et al., 2020). Key metabolic genes involved in oxidative phosphorylation are epigenetically silenced in M2-like TAMs, while glycolytic genes show increased accessibility and expression (Jiang et al., 2024). This metabolic shift reflects a distinct epigenetic program orchestrated by specific histone modifications, including H3K4me1 marks at glycolytic gene enhancers and H3K27me3 marks at oxidative metabolism gene promoters (Saeed et al., 2014).

The metabolic reprogramming of TAMs creates a self-reinforcing loop that maintains their immunosuppressive phenotype. Enhanced glycolysis in TAMs leads to lactate production, which acts through GPR81 receptors to induce further epigenetic changes, including increased expression of HDAC enzymes (Zhang et al., 2022). These HDACs deacetylate histones at pro-inflammatory gene loci, suppressing the production of anti-tumor cytokines and chemokines. Additionally, metabolite-sensitive epigenetic enzymes, such as α-ketoglutarate-dependent dioxygenases, are influenced by the altered metabolic state of TAMs, affecting DNA and histone demethylation processes (Yu and Li, 2024; Lin et al., 2015).

Targeting the epigenetic-metabolic axis in TAMs offers promising strategies for enhancing vaccine responses. Combination approaches using HDAC inhibitors with metabolic modulators can reprogram TAMs toward anti-tumor phenotypes. For instance, inhibiting glycolysis while simultaneously modulating epigenetic enzymes can break the immunosuppressive feedback loop, restoring TAM inflammatory functions and enhancing their capacity to support vaccine-induced T cell responses (Jeong et al., 2019).

### Epigenetic regulation of T lymphocytes

The epigenetic landscape of tumor-infiltrating T lymphocytes profoundly influences their metabolic programming and functional capacity (Liu X. et al., 2022). Effector T cells require robust glycolytic metabolism to support their proliferation and cytotoxic functions, yet the lung tumor microenvironment imposes metabolic restrictions that are reinforced by epigenetic modifications (Beckermann et al., 2017). Exhausted T cells exhibit specific methylation patterns at metabolic gene loci, with hypermethylation of glycolytic enzyme promoters and altered chromatin accessibility at mitochondrial biogenesis genes (Franco et al., 2020).

The metabolic-epigenetic interplay in T cells is mediated by metabolite availability and enzymatic activity. S-adenosylmethionine (SAM), the universal methyl donor, links one-carbon metabolism to DNA and histone methylation (Lee et al., 2023). In the nutrient-depleted tumor microenvironment, altered SAM availability affects methylation patterns, influencing T cell differentiation and function. Similarly, acetyl-CoA levels, determined by the balance between glycolysis and fatty acid oxidation, regulate histone acetylation and gene expression programs in T cells (Soriano-Baguet and Brenner, 2023).

Recent studies have revealed that metabolic interventions can reverse epigenetic T cell dysfunction (Liu et al., 2025a; Han et al., 2023). Supplementation with specific metabolites or use of metabolic pathway inhibitors can restore epigenetic marks associated with effector function. For example, inhibiting lactate dehydrogenase not only reduces lactate production but also alters the NAD^+^/NADH ratio, affecting the activity of sirtuins and other NAD^+^-dependent epigenetic enzymes (Anderson et al., 2017; Xie et al., 2020). This metabolic-epigenetic reprogramming can enhance T cell responses to cancer vaccines by restoring effector functions and preventing exhaustion.

### Epigenetic regulation of cancer-associated fibroblasts

Cancer-associated fibroblasts (CAFs) represent a critical stromal component that undergoes significant epigenetic reprogramming in lung tumors (Raaijmakers et al., 2024). The transformation of normal fibroblasts to CAFs involves widespread changes in DNA methylation and histone modifications that lock in their activated, pro-tumorigenic phenotype (Yang et al., 2023). These epigenetic changes directly influence CAF metabolism, shifting them toward glycolytic metabolism and enhanced production of metabolites that support tumor growth and immunosuppression.

CAFs exhibit unique metabolic features, including reverse Warburg metabolism, where they provide lactate and other metabolites to fuel tumor cells (Liang et al., 2022). This metabolic phenotype is maintained by epigenetic modifications at key metabolic gene loci. Hypomethylation of glycolytic enzyme promoters and altered histone acetylation patterns at oxidative metabolism genes create a stable metabolic program (Kim et al., 2022). Additionally, CAFs produce metabolites that function as epigenetic modifiers, including α-ketoglutarate, succinate, and fumarate, which influence the activity of demethylases in neighboring cells (Mishra et al., 2019).

The secretome of epigenetically reprogrammed CAFs profoundly impacts vaccine responses. CAF-derived factors, including TGF-β, IL-6, and various chemokines, create physical and chemical barriers to T cell infiltration and function (Wu et al., 2021). Epigenetic targeting of CAFs, particularly through DNMT or HDAC inhibition, can normalize their phenotype and reduce their immunosuppressive effects (Ramaiah et al., 2021). Combined with metabolic interventions, epigenetic CAF reprogramming represents a promising strategy for improving vaccine efficacy in lung tumors.

## Nanoplatforms: overcoming the pharmacological barriers of epigenetic therapy

### Epigenetic regulation in lung cancer therapy

Despite the significant potential of epigenetic therapies such as DNMTi and HDACi in lung cancer immunotherapy—including mechanisms like inducing viral mimicry and enhancing antigen presentation—their clinical translation faces considerable pharmacological challenges. These challenges primarily involve severe off-target effects and dose-limiting toxicities (for example myelosuppression) due to their broad mechanisms of action, as well as suboptimal pharmacokinetic properties such as rapid systemic clearance and poor penetration into deep tumor tissues. Furthermore, significant inter- and intratumoral heterogeneity in epigenetic states and immune microenvironments in lung cancer leads to unpredictable and inconsistent treatment responses. These fundamental pharmacological and biological barriers collectively prevent free drugs from achieving and sustaining therapeutically effective concentrations within tumors, thereby hindering durable remodeling of the epigenetic landscape and ultimately limiting the clinical efficacy of both monotherapy and combination strategies with immune checkpoint inhibitors.

Epigenetic therapies have emerged as promising approaches for reversing the profound immune dysregulation in lung tumors. DNA methyltransferase inhibitors (DNMTi) and histone deacetylase inhibitors (HDACi) demonstrate multifaceted mechanisms of action that extend beyond direct cytotoxicity to include robust immunomodulatory effects (Luszczek et al., 2010; Blagitko-Dorfs et al., 2019; Huang et al., 2024). Notably, azacitidine and decitabine induce viral mimicry responses through endogenous retroviral element reactivation, enhancing type I interferon signaling and antigen presentation machinery (Table 1).

**TABLE 1:** Monotherapy in clinical studies.

Drug name	Clinical trial number	Trial phase	Treatment regimen	Lung cancer type
Azacitidine	NCT02009436	Phase II	Monotherapy (inhalation)	Stage IV/Recurrent NSCLC
Decitabine	NCT05960773	Phase II	Monotherapy	BAP1-related early-stage mesothelioma
Vorinostat	NCT00821951	Phase II	Monotherapy combined with palliative radiotherapy	NSCLC
Vorinostat	NCT00667082	Phase I	Combination with NPI-0052 (Marizomib)	NSCLC and others
Panobinostat	NCT01222936	Phase II	Monotherapy	SCLC
Belinostat	NCT00926640	Phase I	Combination with cisplatin + etoposide	SCLC

Yet, the clinical translation of these agents has been tempered by significant clinical hurdles, starkly illustrating the discrepancy between preclinical potential and clinical reality. The trials of epigenetic monotherapies are paradigmatic. For instance, the phase II study of systemically administered Vorinostat with radiotherapy (NCT00821951) failed to yield breakthroughs in NSCLC. This outcome is largely attributed to its narrow therapeutic window; the doses required to avoid systemic toxicities like fatigue and thrombocytopenia are likely insufficient to achieve the sustained, biologically effective concentrations needed within the TME to durably remodel the epigenetic landscape and reverse T-cell exhaustion. This limitation persists even when attempting to bypass systemic routes. An innovative trial exploring inhaled Azacitidine (NCT02009436) also met with limited success, suggesting that overcoming systemic toxicity is only half the battle. The trial’s failure underscores that free drugs, even when delivered locally, face formidable intratumoral barriers, including rapid clearance and poor penetration through dense stromal architecture.

The strategy of combining epigenetic agents with immune checkpoint inhibitors (ICIs) has yielded encouraging signals (Table 2), yet the clinical trials themselves have uncovered profound, unresolved complexities that temper enthusiasm and guide future research. For example, the phase II study of Azacitidine plus Nivolumab (NCT02546986), while demonstrating some clinical activity, produced a modest objective response rate (ORR). A crucial lesson from this trial is the decisive role of patient heterogeneity. The study did not employ biomarker-based patient stratification, such as pre-treatment DNA methylation profiles or baseline immune infiltration status, leaving a critical question unanswered: which patient subgroups are most likely to benefit from this dual strategy of ‘epigenetic reprogramming’ and ‘immune checkpoint liberation’? This highlights the paramount urgency for developing robust predictive biomarkers.

**TABLE 2:** Clinical combinations with immunotherapies.

Drug name	Clinical trial number	Trial phase	Treatment regimen	Lung cancer type
Azacitidine	NCT02959437	Phase I	Pembrolizumab	Advanced solid tumors including NSCLC
Azacitidine	NCT02546986	Phase II	Nivolumab	NSCLC
Decitabine	NCT02664181	Phase I	Nivolumab	NSCLC
Vorinostat	NCT02638090	Phase I	Pembrolizumab	NSCLC
Entinostat	NCT01928576	Phase II	Nivolumab	NSCLC
Mocetinostat	NCT02805660	Phase I	Durvalumab	Advanced solid tumors including NSCLC
Tazemetostat	NCT05353439	Phase I	Pembrolizumab	Recurrent SCLC
Tazemetostat	NCT05467748	Not Specified	Pembrolizumab	NSCLC
XNW5004	NCT06022757	Phase I	Pembrolizumab	Advanced solid tumors including lung cancer

Complementing the challenge of patient selection is the equally critical issue of therapeutic scheduling and dynamics. While the combination of Entinostat and Nivolumab (NCT01928576) produced encouraging results, the dosing and timing regimens were largely empirical. We lack a fundamental understanding of the optimal ‘time window’ for epigenetic-drug-induced antigen expression and immune cell reprogramming. Should treatment involve a prolonged, low-dose ‘epigenetic priming’ to ‘warm up’ the TME before ICI administration, or is a concurrent, high-dose pulse more effective? This challenge of dynamic therapeutic optimization represents a significant, yet largely overlooked, scientific frontier that current clinical trial designs have not systematically addressed.

Collectively, these trials underscore that the next-generation of combination therapies must evolve beyond simply mixing active agents and towards a sophisticated, biomarker-guided approach that personalizes treatment to both the patient and the dynamic temporal evolution of the tumor-immune dialogue. The clinical setbacks are rooted in a confluence of fundamental pharmacological and biological barriers (Zhou et al., 2023). The non-specific mechanism of action of current epigenetic drugs results in substantial off-target effects and dose-limiting toxicities like myelosuppression, while their suboptimal pharmacokinetic properties are characterized by rapid clearance and poor tissue penetration (Xu et al., 2023). Compounding these issues is the profound epigenetic and immunological heterogeneity across lung tumors, which dictates differential therapeutic responses. It is precisely this multifaceted challenge—requiring therapies that can navigate systemic toxicities, breach physical tumor barriers, and be deployed with precise temporal control—that nanoplatform-based delivery systems are poised to address. Advanced nanocarriers offer the potential to resolve these limitations by simultaneously widening the therapeutic window, overcoming delivery barriers, and enabling the spatiotemporal control required to orchestrate a productive anti-tumor immune response.

Cancer vaccination in lung tumors depends on a functional cancer-immunity cycle (CI cycle), which requires robust antigenicity and adjuvanticity to sustain antitumor immunity. The cycle involves seven steps: antigen release and capture, processing and presentation, T-cell priming, trafficking, infiltration, tumor recognition, and killing. However, epigenetic and metabolic dysregulation disrupts multiple stages beyond adjuvanticity. For example, DNMT-mediated hypermethylation silences tumor antigens (for example MAGE-A3), while lactate accumulation in the TME inhibits TET demethylases in dendritic cells, impairing antigen presentation. IDO-driven tryptophan catabolism enhances EZH2 activity in T cells, repressing effector genes via H3K27me3 and promoting T-cell anergy. CAFs further disrupt T-cell trafficking through epigenetic silencing of chemokines like CXCL10. Nanomaterials can simultaneously target these barriers: pH-responsive nanoparticles co-delivering DNMT and LDHi restore antigen expression, improve DC function, and enhance T-cell activation, as shown by increased CD8^+^ T-cell infiltration and tumor control in preclinical models. A broader CI cycle-focused approach is essential to improve response rates in lung cancer vaccines (Liu et al., 2024).

### Targeted nano-delivery systems

**FIGURE 4:**
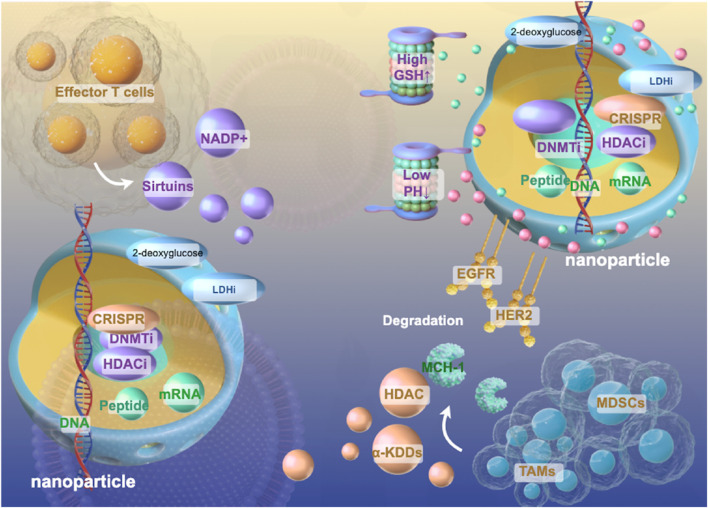
Schematic Diagram of Epigenetics-Centered Multifunctional Nanoplatform Design. Multifunctional nanoplatforms integrating epigenetic therapeutics (DNMTi, HDACi, CRISPR components) with metabolic modulators (2-deoxyglucose, LDHi) to synergistically reprogram the immunosuppressive lung tumor microenvironment as described in sections 5.2–5.3. These sophisticated delivery systems incorporate tumor-targeting ligands (EGFR, HER2) and stimuli-responsive elements (pH, GSH-sensitive) that enable precise spatiotemporal control of drug release within the complex immunosuppressive cellular architecture comprising effector T cells, MDSCs, and TAMs. By simultaneously disrupting the self-reinforcing epigenetic-metabolic feedback loops that maintain immunosuppressive phenotypes while enhancing antigen presentation machinery, these nanoplatforms represent a promising strategy to overcome the formidable barriers to cancer vaccine efficacy in lung tumors through comprehensive reprogramming of the tumor immune landscape.

The integration of epigenetic modulators into nanoplatform designs could potentially revolutionize approaches to lung cancer therapy. Nanocarriers offer unique advantages for delivering epigenetic drugs, including protection from degradation, enhanced tumor accumulation, and controlled release kinetics. Lipid-based nanoparticles have shown particular promise for delivering DNMTi and HDAC inhibitors, with modifications such as PEGylation extending circulation time and reducing immunogenicity (Sukocheva et al., 2022) (Figure 4).

Advanced nanoplatform designs incorporate stimuli-responsive elements that enable precise spatiotemporal control of epigenetic drug release. pH-responsive nanocarriers exploit the acidic tumor microenvironment to trigger drug release specifically within tumors (Chen et al., 2023). Redox-responsive systems utilize the elevated glutathione levels in cancer cells to achieve intracellular drug release (Raza et al., 2018). These smart delivery systems minimize off-target effects while maximizing therapeutic efficacy.

Cell-specific delivery to immune populations within the tumor microenvironment requires sophisticated targeting approaches (Lei et al., 2020). Nanoparticles decorated with antibodies against immune cell markers can selectively deliver cargo to specific immune subsets (Kimmel et al., 2025; Jain et al., 2024). For instance, CD3e f (ab)2 fragment nanoparticles can deliver metabolic modulators specifically to cytotoxic T cells (Kim et al., 2021), while anti-F4/80 targeting enables macrophage-specific delivery (Terry et al., 2015). This precision targeting minimizes systemic effects while maximizing local immunomodulation.

The tumor microenvironment presents unique opportunities for environmental targeting. Enzyme-cleavable linkers responsive to matrix metalloproteinases enable selective drug release in the tumor stroma (Li et al., 2020; Iaccarino et al., 2019). Hypoxia-responsive nanocarriers utilize the low oxygen tension in tumors to trigger drug release through reduction of azobenzene or nitroimidazole groups (Thambi et al., 2014). These environmental targeting strategies complement receptor-based approaches to achieve optimal drug delivery.

Preclinical studies investigating epigenetic modulator-loaded nanoplatforms have established compelling proof-of-concept, as summarized in Table 3. Lipid-based nanoformulations, for example, have shown considerable promise by enabling the co-delivery of synergistic epigenetic agents, such as decitabine and panobinostat, to enhance anti-tumor efficacy in preclinical models (Rehman et al., 2024). These advances, marked by favorable biodistribution and enhanced anti-tumor immune responses, are certainly encouraging. However, these promising findings must be interpreted with caution, as a critical appraisal reveals significant translational challenges embedded within the study designs.

**TABLE 3:** Applications of epigenetic modification combined with nanotechnology.

Drug name	Nano carrier type	Lung cancer animal model	Main results
Vorinostat (Sankar and Ravikumar, 2014)	PLGA polymeric nanoparticles	*In vitro* and *in vivo* A549 lung cancer cell models	PLGA nanoparticles loaded with Vorinostat showed good biocompatibility and biodistribution, and were actively taken up by A549 lung cancer cells
Vorinostat (Shanmugam et al., 2022)	PLGA nanoparticles	Lung cancer cell models	The nanoparticles exhibited enhanced permeability and retention (EPR) effect, showing active uptake and favorable biodistribution patterns in lung cancer cells and tumor models
GSK126 (Guo et al., 2025)	Albumin nanoparticles (GSK126 NPs)	B16F10 melanoma xenograft mouse model	Significantly reduced tumor weight and volume with no obvious systemic toxicity; partially improved the induction effect of GSK126 on MDSCs
EZH2 siRNA (Wang et al., 2019)	DMC nanocomplex	BALB/c female nude mouse orthotopic U87 glioma model	The EZH2si-DMC complex more effectively inhibited tumor growth than other groups, and the mice in the treatment group had the longest survival
EZH2 siRNA (Lu et al., 2023)	Magnetic nanodrug carrier	Triple-negative breast cancer mouse model	Combination of chemotherapy and gene therapy significantly increased tumor inhibition effect, showing good safety characteristics
Vorinostat (Kwak et al., 2015)	Polymeric nanoparticles	HuCC-T1 cholangiocarcinoma xenograft nude mouse model	The nanoformulation showed stronger antitumor activity than the free drug; drug retention time at the tumor site was extended to 8 days
Entinostat (Abed et al., 2024)	Polymeric nanoparticles	Colorectal cancer cell models	Maintained drug activity, and combined with MDM2 inhibitor RG7388 showed a synergistic effect in inducing cell death
Quisinostat (Wang et al., 2015)	Nanoparticle formulation	Mouse xenograft models	As a radiotherapy sensitizer, it showed better efficacy than small-molecule drugs
HDACi 4b (Jia et al., 2015)	Unspecified carrier	Huntington’s disease R6/2 mouse model	Improved body weight and motor function, reduced brain atrophy, and at least partial recovery of expression in 90% of affected genes
Azacitidine (Jahanfar et al., 2016)	Solid lipid nanoparticles (SLNs)	MCF-7 breast cancer cell line	The encapsulated drug showed significantly higher cytotoxicity than the free drug; induced morphological changes of apoptosis; promoted RARβ2 gene expression
Azacitidine (Elzayat et al., 2023)	Lipid nanoparticles (GEF-AZT-NLC)	Metastatic drug-resistant lung cancer model	Significantly improved cell uptake efficiency and cell killing effect
Decitabine (Wu et al., 2017)	Bone-targeted nanoparticles (BTNPs)	NUP98/HOXD13 transgenic mouse MDS model	Significantly improved hematological parameters and reduced toxicities such as thrombocytopenia and leukopenia
Azacitidine (Mitsuhashi et al., 2025)	PLGA core-lipid shell hybrid carrier	HCT116 colorectal cancer cells	Dual targeting of DNMT and TET enzymes, effectively repairing abnormal DNA methylation and inducing G2/M phase cell cycle arrest

To begin with, the choice of animal model often inflates therapeutic expectations. The success of nanoparticles in an immunologically favorable model, which is inherently sensitive to immunotherapy, cannot be directly extrapolated to the profoundly immunosuppressive microenvironment of primary lung cancer (Wang et al., 2024). To generate more predictive data, future preclinical validation must pivot towards more clinically relevant systems, such as Kras/p53 genetically engineered mouse models or patient-derived xenografts (PDX) (Nakahata et al., 2022). Furthermore, the concept of ‘targeted delivery’ itself warrants critical scrutiny. Despite reports of sophisticated targeting strategies, the unavoidable reality is that a majority of nanoparticles are sequestered by the reticuloendothelial system (RES) (Tang et al., 2019). This ‘off-target’ accumulation is not merely a loss of payload but a potentially potent immunomodulatory event—for instance, by altering Kupffer cell function or systemic T-cell priming—a “double-edged sword” effect that remains a largely unexplored dimension of nanomedicine. Finally, the very elegance of these nanoplatforms often conceals their greatest translational barrier: manufacturing complexity (Feng et al., 2024). The chemistry, manufacturing, and controls (CMC) for multi-component systems are exceptionally demanding (O'Brien Laramy et al., 2025). A forward-looking perspective must therefore recognize that the next breakthrough in this field may lie not in increasing design complexity, but in mastering the manufacturability and scalability required for clinical translation.

### Synergistic effects of co-loading metabolic regulators and epigenetic nanomedicine

The co-encapsulation of metabolic regulators and epigenetic drugs within nanoplatforms may generates synergistic effects that extend far beyond simple additive responses (Liu S. et al., 2025; Zhou Y. et al., 2024). This synergy arises from the fundamental interconnection between cellular metabolism and epigenetic regulation, where metabolites serve as essential cofactors for epigenetic enzymes while epigenetic modifications control the expression of metabolic genes (Thakur and Chen, 2019). The simultaneous modulation of both systems creates a powerful positive feedback loop that amplifies therapeutic efficacy.

The powerful synergy generated by co-encapsulating metabolic regulators and epigenetic drugs can be understood rationally through the lens of the Epi-Met-Immune Network. Rather than being a simple additive effect, this strategy represents a concerted attack on the feedback loops that maintain immunosuppression. For example, by simultaneously delivering a glycolytic inhibitor and an HDACi, a nanoplatform can disrupt both a key node in the Metabolic Layer (lactate production) and another in the Epigenetic Layer (histone acetylation), effectively dismantling the self-reinforcing circuit that connects metabolic hostility to T-cell epigenetic silencing.

At the molecular level, metabolic inhibitors such as 2-deoxyglucose or lactate dehydrogenase inhibitors reduce the production of oncometabolites that normally inhibit epigenetic enzymes (Wong et al., 2017). For instance, decreased lactate production enhances the activity of histone deacetylases by altering the NAD^+^/NADH ratio, while reduced 2-hydroxyglutarate levels restore the function of TET enzymes and histone demethylases (An et al., 2023). When combined with direct epigenetic modulators like HDAC inhibitors or DNA methyltransferase inhibitors, this metabolic reprogramming synergistically enhances chromatin remodeling and gene expression changes (Ramaiah et al., 2021). Studies have demonstrated that this combination achieves greater changes in immune-related gene expression compared to either treatment alone (Liang et al., 2023; Fang et al., 2021).

This combination effect may have a significant impact on immune cell function within the tumor microenvironment. Co-delivery of glycolytic inhibitors with epigenetic drugs not only reduces metabolic competition between tumor cells and T cells but also prevents the epigenetic imprinting of exhaustion programs (Geng et al., 2023). This action results in a enhancement in T cell cytotoxicity compared to monotherapy approaches.

The temporal dynamics of synergistic effects reveal another layer of complexity. Metabolic reprogramming can sensitize cells to subsequent epigenetic interventions by altering the availability of metabolic cofactors (Sun et al., 2022). S-adenosylmethionine levels, modulated by methionine metabolism inhibitors, directly influence DNA and histone methylation patterns (Pascale et al., 2022). When combined with DNMT inhibitors, this metabolic priming enhances demethylation efficiency (Blagitko-Dorfs et al., 2019). Conversely, epigenetic drugs can reprogram metabolic gene expression, creating sustained metabolic changes that persist beyond drug clearance (Peng and Zhong, 2015). This bidirectional enhancement creates durable therapeutic effects that extend the duration of immune activation.

The synergy extends to overcoming drug resistance mechanisms. Tumor cells often develop resistance to metabolic inhibitors through compensatory metabolic pathways, but co-delivered epigenetic drugs can silence these escape routes by modulating the expression of alternative metabolic enzymes (Park et al., 2020; Scumaci and Zheng, 2023). Similarly, epigenetic drug resistance mediated by drug efflux pumps or metabolic inactivation can be circumvented by metabolic modulators that alter cellular energy states and transporter function (Ingelman-Sundberg et al., 2013). This reciprocal resistance prevention has been demonstrated to maintain drug sensitivity longer than single-agent treatments in preclinical models (Singh and Yeh, 2017).

### Immune adjuvant functions of epigenetic nano-vaccines

Beyond drug delivery, nanoplatforms themselves can function as immune adjuvants, enhancing vaccine responses through multiple mechanisms (Zhao T. et al., 2023). The physicochemical properties of nanoparticles, including size, shape, and surface chemistry, influence their immunogenicity (David et al., 2016; Lin et al., 2020). Nanoparticles in the 20–200 nm range are efficiently taken up by dendritic cells and transported to lymph nodes, optimal for initiating immune responses (Manolova et al., 2008; Zhao H. et al., 2023). Surface modifications with pathogen-associated molecular patterns (PAMPs) further enhance their adjuvant activity (Ben-Akiva et al., 2025).

Inorganic nanoparticles, particularly those based on gold, silica, or iron oxide, can activate innate immune responses through multiple pathways (Palomino-Cano et al., 2024). These materials can trigger inflammasome activation, leading to IL-1β production and enhanced antigen presentation (van de Veerdonk et al., 2011). The controlled release of ions from degradable inorganic nanoparticles provides sustained immune stimulation (Liu et al., 2025c). Additionally, the photothermal properties of certain nanoparticles enable combination with thermal ablation therapies, releasing tumor antigens while providing adjuvant signals (Ashikbayeva et al., 2019).

Biomimetic nanoplatforms represent an emerging frontier in vaccine design (Liu J. et al., 2022). Cell membrane-coated nanoparticles combine the drug delivery capabilities of synthetic carriers with the biological functions of cell membranes (Xu et al., 2020). Tumor cell membrane-coated particles present a full array of tumor antigens while protecting encapsulated drugs (Jiang et al., 2020). Dendritic cell membrane coatings provide natural targeting to lymph nodes and enhanced T cell activation (Cao et al., 2023). These biomimetic approaches blur the lines between drug delivery vehicles and vaccines themselves.

## Future directions and clinical translation challenges

### From single-node targeting to rational network disruption

The future of epigenetic nanomedicine lies not in simply improving the delivery of single agents, but in rationally designing platforms that can overcome the TME’s most formidable property: its capacity for adaptive resistance. This requires elevating our view of the Epi-Met-Immune Synergistic Network from a static map of immunosuppression to a dynamic engine of therapeutic failure. When a single node is targeted with a monotherapy, such as an EZH2 inhibitor, the network often responds not by collapsing, but by adaptively rewiring itself. The system compensates by upregulating alternative metabolic pathways or engaging different epigenetic silencing mechanisms, effectively circumventing the therapeutic blockade and driving resistance.

This inherent resilience renders single-agent strategies fundamentally inadequate and reframes the mission of nanomedicine: the goal is not merely to achieve synergy, but to preemptively dismantle the network’s capacity for adaptive resistance. This is the ultimate rationale for multi-pronged, systems-level disruption. The next paradigm shift will involve leveraging patient-specific data to guide these attacks. For instance, emerging liquid biopsy technologies that map circulating tumor DNA methylation patterns will not only serve as diagnostic biomarkers but will also reveal the network’s active pathways and predict its likely escape routes (Luo et al., 2021). This information, when processed by AI-driven algorithms, can guide the selection or even *de novo* design of a nanoplatform co-delivering a specific combination of agents—such as an EZH2 inhibitor and a lactate dehydrogenase inhibitor—to sever not only the primary driver pathways but also the anticipated resistance circuits (Wei et al., 2021; Zhang et al., 2017). This represents the ultimate goal of precision medicine: moving from pathway-level intervention to patient-specific network demolition.

### A clinical roadmap: from network theory to precision intervention

**FIGURE 5:**
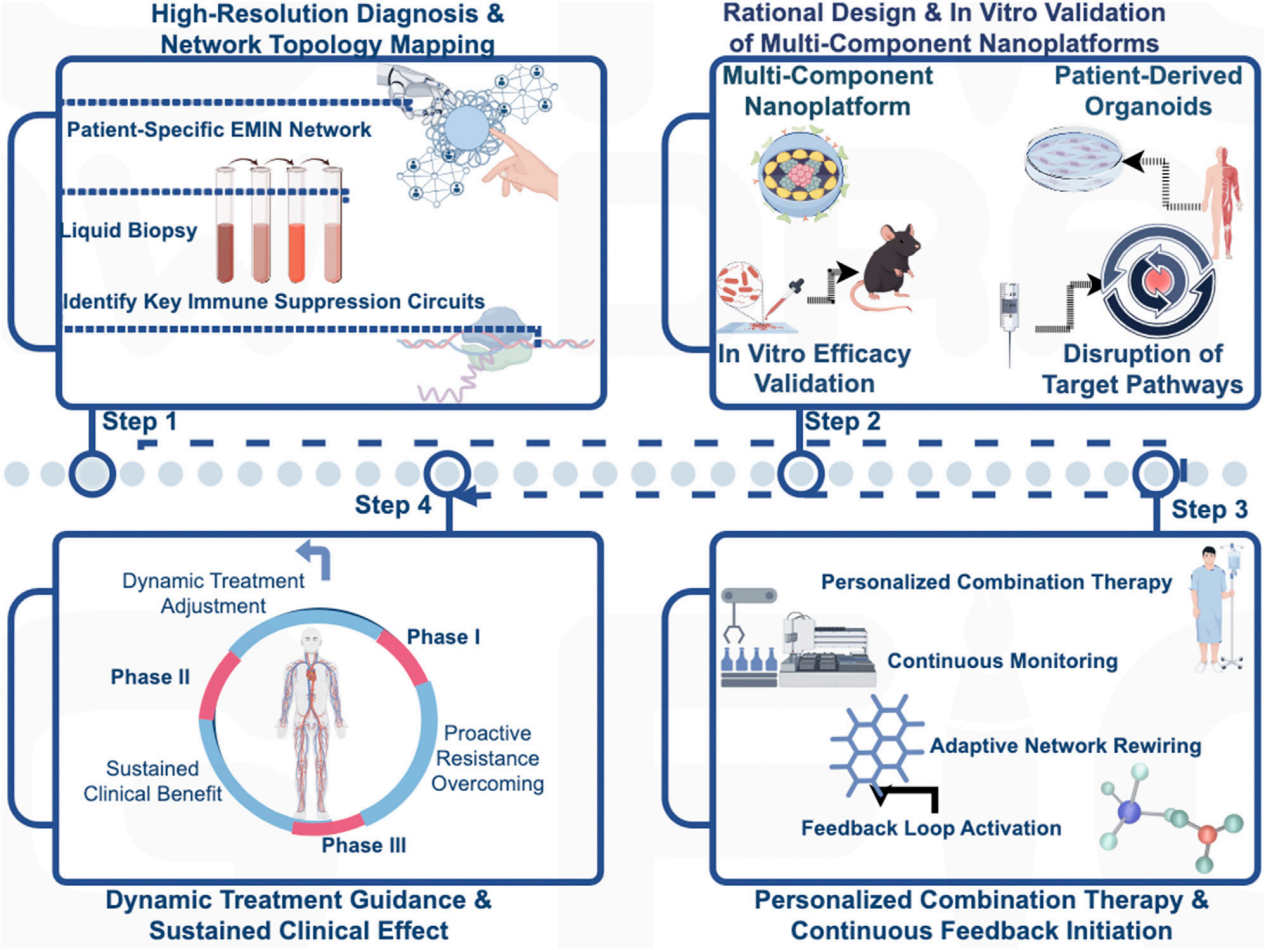
A Closed-Loop Clinical Paradigm for Precision Intervention Based on the Epi-Met-Immune Network. This figure illustrates a proposed closed-loop, four-stage clinical paradigm for operationalizing the Epi-Met-Immune Network concept to guide personalized cancer therapy. (Stage 1: High-Resolution Diagnosis) The process begins with a high-resolution diagnosis, utilizing technologies such as liquid biopsy to map the patient-specific network topology and identify the dominant immunosuppressive circuits. (Stage 2: *Ex Vivo* Validation) Based on this functional map, a rationally selected multi-component nanoplatform is subjected to rigorous *ex vivo* validation in patient-derived models, such as tumor organoids, to confirm its ability to dismantle the identified target pathways. (Stage 3: Personalized Administration) Only after this personalized confirmation of efficacy is the synergistic therapy administered to the patient, initiating a continuous feedback loop. (Stage 4: Dynamic Monitoring and Steering) Finally, serial monitoring is employed to track the network’s adaptive rewiring in real-time, enabling dynamic therapeutic steering to preemptively counter resistance and ensure durable clinical benefit.

Operationalizing the Epi-Met-Immune Network concept requires a closed-loop, four-stage clinical paradigm. The process initiates with a high-resolution diagnosis, using liquid biopsies to map the patient-specific network topology and identify its dominant immunosuppressive circuits. This functional map then guides the rigorous *ex vivo* validation of a rationally selected multi-component nanoplatform in patient-derived models, such as tumor organoids, to confirm its ability to dismantle the target pathways. Only upon this personalized confirmation of efficacy is the synergistic therapy administered, initiating a continuous feedback loop where serial monitoring tracks the network’s adaptive rewiring in real-time. This final stage enables dynamic therapeutic steering, allowing for the adjustment of treatment to preemptively counter resistance and ensure durable clinical benefit (Figure 5).

### Overcoming the physical and manufacturing barriers

Despite promising preclinical results, the translation of these sophisticated network-disrupting therapies faces two intertwined engineering challenges: manufacturing scalability and penetrating the tumor microenvironment (Metselaar and Lammers, 2020). The clinical translation of multi-component nanotherapeutics is critically bottlenecked by challenges in CMC, where establishing standardized, scalable processes that ensure batch-to-batch consistency and long-term stability is paramount (Gawne et al., 2023). This complexity is mirrored by regulatory hurdles, as agencies like the FDA and EMA require stringent characterization and safety assessments, making early and continuous engagement essential. Once successfully manufactured, these nanoparticles confront the second challenge: the profound heterogeneity of the TME, which severely limits the universal applicability of passive targeting via the EPR effect (Du et al., 2015). Successfully delivering a network-disrupting payload requires overcoming formidable physical barriers—including anomalous vasculature and a rigid extracellular matrix—and hostile chemical gradients like hypoxia and acidity (Ross et al., 2015). Therefore, future strategies must evolve beyond passive accumulation to include active targeting ligands, biomimetic coatings that use immune cells as Trojan horses, and intelligent, stimuli-responsive systems designed to trigger drug release only upon reaching the specific metabolic or pH conditions of the deep tumor core.

### Ensuring clinical viability and economic accessibility

Ultimately, the success of these transformative therapies will be determined by their real-world clinical viability and economic sustainability. While the initial investment for advanced nanomedicines is substantial, their potential to offer durable responses or even cures provides considerable long-term economic value by reducing downstream healthcare costs and enhancing patient productivity (Bosetti and Jones, 2019). However, realizing this potential requires a paradigm shift in implementation. This includes adopting value-based pricing models that link payment to clinical outcomes, exploring innovative financing mechanisms, and integrating cost-effectiveness analyses early in the development process. Ensuring equitable access to these technologies will require a comprehensive framework that balances immediate budgetary constraints with long-term societal benefit, making these powerful network-disrupting therapies a reality for patients (Toro et al., 2025).

## Conclusion

The convergence of nanotechnology with epigenetic and metabolic modulation represents a transformative frontier in cancer immunotherapy. This review has advanced the concept that durable anti-tumor immunity is hindered not by isolated pathways but by a resilient, interconnected network of epi-metabolic feedback loops. By proposing the Epi-Met-Immune Synergistic Network as a conceptual framework, we provide a rational basis for designing sophisticated nanoplatforms capable of systems-level intervention—co-delivering synergistic agents to dismantle the very foundations of immunosuppression. While overcoming translational hurdles in manufacturing and delivery remains critical, this network-guided approach promises to fundamentally reshape cancer vaccine development, transforming immunologically “cold” tumors into responsive malignancies amenable to precision therapy.
